# Timing of introduction to solid food, eczema and wheezing in later childhood: a longitudinal cohort study

**DOI:** 10.1186/s12887-023-04262-0

**Published:** 2023-10-16

**Authors:** Curtis J. D’Hollander, Charles D. G. Keown-Stoneman, Catherine S. Birken, Deborah L. O’Connor, Jonathon L. Maguire, TARGet Kids! collaboration, Christopher Allen, Laura N. Anderson, Danielle D’Annunzio, Mateenah Jaleel, Natricha Levy McFarlane, Jessica A. Omand, Sharon Thadani, Mary Aglipay, Imaan Bayoumi, Cornelia M. Borkhoff, Sarah Carsley, Alice Charach, Katherine Cost, Anne Fuller, Laura Kinlin, Michaela Kucab, Patricia Li, Pat Parkin, Nav Persaud, Sarah Rae, Izabela Socynska, Shelley Vanderhout, Leigh Vanderloo, Peter Wong, Piyumi Konara Mudiyanselage, Xuedi Li, Jenny Liu, Michelle Mitchell, Yulika Yoshida-Montezuma, Nusrat Zaffar, Tiffany Bondoc, Trudy-Ann Buckley, Pamela Ruth Flores, Kardelen Kurt, Sangeetha Loganathan, Tarandeep Mali, Laurie Thompson, Jennifer Batten, Jennifer Chan, John Clark, Amy Craig, Kim De Castris-Garcia, Sharon Dharman, Sarah Kelleher, Salimah Nasser, Tammara Pabon, Michelle Rhodes, Rafael Salsa, Julie Skelding, Daniel Stern, Kerry Stewart, Erika Sendra Tavares, Shannon Weir, Maria Zaccaria Cho, Magdalena Janus, Eric Duku, Caroline Reid-Westoby, Patricia Raso, Amanda Offord, Emy Abraham, Sara Ali, Kelly Anderson, Gordon Arbess, Jillian Baker, Tony Barozzino, Sylvie Bergeron, Gary Bloch, Joey Bonifacio, Ashna Bowry, Caroline Calpin, Douglas Campbell, Sohail Cheema, Elaine Cheng, Brian Chisamore, Evelyn Constantin, Karoon Danayan, Paul Das, Viveka De Guerra, Mary Beth Derocher, Anh Do, Kathleen Doukas, Anne Egger, Allison Farber, Amy Freedman, Sloane Freeman, Sharon Gazeley, Karen Grewal, Charlie Guiang, Dan Ha, Curtis Handford, Laura Hanson, Leah Harrington, Sheila Jacobson, Lukasz Jagiello, Gwen Jansz, Paul Kadar, Lukas Keiswetter, Tara Kiran, Holly Knowles, Bruce Kwok, Piya Lahiry, Sheila Lakhoo, Margarita Lam-Antoniades, Eddy Lau, Denis Leduc, Fok-Han Leung, Alan Li, Patricia Li, Roy Male, Aleks Meret, Elise Mok, Rosemary Moodie, Katherine Nash, James Owen, Michael Peer, Marty Perlmutar, Nav Persaud, Andrew Pinto, Michelle Porepa, Vikky Qi, Noor Ramji, Danyaal Raza, Katherine Rouleau, Caroline Ruderman, Janet Saunderson, Vanna Schiralli, Michael Sgro, Hafiz Shuja, Farah Siam, Susan Shepherd, Cinntha Srikanthan, Carolyn Taylor, Stephen Treherne, Suzanne Turner, Fatima Uddin, Meta van den Heuvel, Thea Weisdorf, Peter Wong, John Yaremko, Ethel Ying, Elizabeth Young, Michael Zajdman, Esmot Ara Begum, Gurpreet Lakhanpal, Gerald Lebovic, Ifeayinchukwu (Shawn) Nnorom, Marc Denzel Nunez, Audra Stitt, Kevin Thorpe, Raya Assan, Homa Bondar, George Charames, Andrea Djolovic, Chelseas Gorscak-Dunn, Mary Hassan, Rita Kandel, Michelle Rodrigues

**Affiliations:** 1 Department of Nutritional Sciences, University of Toronto, Toronto, ON Canada; 2 Department of Pediatrics, St Michael’s Hospital, Toronto, ON Canada; 3 Applied Health Research Centre, Unity Health Toronto, Toronto, ON Canada; 4 Dalla Lana School of Public Health, University of Toronto, Toronto, ON Canada; 5 Division of Pediatric Medicine, Hospital for Sick Children, Toronto, ON Canada; 6 Child Health Evaluative Sciences, SickKids Research Institute, Toronto, ON Canada; 7 Department of Pediatrics, Faculty of Medicine, University of Toronto, Toronto, ON Canada; 8 Institute of Health Policy Management and Evaluation, University of Toronto, Toronto, ON Canada; 9 Joannah & Brian Lawson Centre for Child Nutrition, Department of Nutritional Sciences, University of Toronto, Toronto, ON Canada; 10 Translational Medicine Program, Hospital for Sick Children, Toronto, ON Canada; 11 Li Ka Shing Knowledge Institute, St. Michael’s Hospital, Toronto, ON, Canada

**Keywords:** Timing of introduction to solid food, Atopic disease, Allergy, Atopic dermatitis, Wheeze, Breastfeeding, Eczema

## Abstract

**Background:**

The timing of introduction to solid food has been associated with eczema and wheezing in childhood. Our aim was to determine whether differences persist within the recommended 4 to 6 month age range.

**Methods:**

A longitudinal cohort study with repeated measures was conducted among children from birth to 10 years of age who were participating in the TARGet Kids! practice based research network in Toronto, Canada. The primary exposure was the timing of introduction to infant cereal as the first solid food. The primary outcome was eczema and the secondary outcome was wheezing collected by parent report using the validated International Study of Asthma and Allergies in Childhood (ISAAC) questionnaire. Multinomial generalized estimating equations were used and effect modification by family history of asthma and breastfeeding duration were explored.

**Results:**

Of the 7843 children included, the mean (standard deviation) age of introduction to infant cereal was 5.7 (1.9) months. There was evidence for family history of asthma and breastfeeding duration to be effect modifiers in the eczema (*P* = 0.04) and wheezing (*P* = 0.05) models. Introduction to infant cereal at 4 vs. 6 months of age was associated with higher odds of eczema (OR 1.62; 95% CI: 1.12, 2.35; *P* = 0.01) among children without a family history of asthma who were not breastfeeding when solid foods were introduced. Introduction to infant cereal at 4 vs. 6 months of age was associated with a higher odds of wheezing (OR 1.31; 95% CI: 1.13, 1.52; *P* < .001) among children without a family history of asthma who were breastfeeding when solid foods were introduced. There was little evidence of an association among the remaining strata for either outcome.

**Conclusion:**

The findings of this study support recommendations to introduce solid food around 6 months of age.

## Background

Eczema is a group of conditions which cause inflamed and irritated skin, and atopic dermatitis (AD) is the most common form of eczema in childhood [1, 2]. AD is a chronic, relapsing inflammatory disease of the skin characterized by an immunoglobulin (IgE) response to allergens [1]. Wheezing may be atopic or non-atopic, and can occur when there is narrowing of the airway due to IgE mediated inflammation [1, 3]. Children with one atopic disease are at high risk for developing another [1, 4]. It has been estimated that 15–20% of children have eczema, and 10–20% have wheezing at some point in childhood [5, 6].

The timing of introduction to solid food may be an important risk factor for the development of eczema and wheezing. The American Academy of Pediatrics (AAP) and Canadian Paediatric Society (CPS) recommend that solid food be introduced around 6 months of age while the European Society for Paediatric Gastroenterology, Hepatology, and Nutrition (ESPGHAN) recommend that solid food be introduced between 4 and 6 months of age [7–9]. Several studies have shown an association between introduction to solid food (i.e. < 4 months or > 6 months of age) and eczema and wheeze [10–13]. For instance, Taylor-Robinson et al. found introduction to solid foods < 4 months, compared to later, to be associated with higher odds of eczema by 5 years of age (OR 1.13; 95% CI: 1.04, 1.23) [10]. Snijders et al. found introduction to solid foods > 7 months, compared to earlier, to be associated with higher odds of eczema (OR 2.10; 95% CI: 1.17, 3.76; *P*-trend = 0.02) and wheeze (OR 3.52; 95% CI: 1.42, 8.73; *P*-trend = 0.01) by 2 years of age [11]. However, the relationship between the timing of introduction to solid food within the recommended 4 to 6 month range and eczema and wheezing is unclear. Known risk factors for eczema and wheezing include a family history of allergies and breastfeeding duration [1, 14, 15].

The primary objective of this study was to evaluate the relationship between the timing of introduction to infant cereal as the first solid food at 4 months compared to 6 months of age and parent reported eczema or AD. The secondary objective was to examine the relationship between the timing of introduction to infant cereal at 4 months compared to 6 months of age and parent report of wheezing. We also explored whether these relationships were modified by family history of asthma and breastfeeding duration.

## Methods

### Study design and participants

A longitudinal cohort study was conducted using repeated measures from birth to 10 years of age among healthy children who were recruited while attending a scheduled primary health care visit at one of ten participating TARGet Kids! primary care practices between September 2011 and August 2019. TARGet Kids! is a primary care, practice-based research network in Toronto, Canada (www.targetkids.ca) [16]. The study protocol has been described in detail elsewhere [16]. Data were collected at regularly scheduled primary healthcare visits approximately twice a year until age 2 years, and then annually until age 10 years in accordance with the CPS schedule of well-child visits. Trained research assistants collected sociodemographic information, health and lifestyle factors, including exposure and outcome data at each visit. Children with health conditions affecting growth (e.g. failure to thrive, cystic fibrosis), chronic conditions (except asthma, eczema, wheezing), those born very premature (< 32 weeks gestation) and children with severe developmental delay at enrollment were excluded from the TARGet Kids! cohort. In addition, children who were not introduced to infant cereal as the first solid food (e.g. only visit occurred prior to introducing solid food or introduced to different solid food) were excluded from this study. All methods were done in accordance with the relevant guidelines and regulations including the Helsinki Declaration on ethical principles. Written informed consent was obtained from parents, and ethical approval granted from the Research Ethics Board at St. Michael’s Hospital and The Hospital for Sick Children.

### Exposure variable

The primary exposure was parent reported timing of introduction of infant cereal as the first solid food. Caregivers completed a nutrition questionnaire adapted from the Canadian Community Health Survey which included the question “At what age did you introduce infant cereal to your child? (months)” [17]. To minimize the duration of recall, timing of introduction to infant cereal was measured at the first visit following introduction to infant cereal. For descriptive analyses, non-integer responses were categorized as follows: responses of ≥ 4 months and < 5 months or ≥ 6 months and < 7 months were categorized as introduction to infant cereal at 4 months and 6 months, respectively. Otherwise, all analyses included timing of introduction to infant cereal as a continuous variable.

### Outcome variables

The primary outcome variable was eczema or AD. Parents completed a questionnaire which included the question “Has your child been diagnosed with Eczema or Atopic Dermatitis? (yes/no).” The secondary outcome was wheezing. Parents were asked “Has your child ever had wheezing or whistling in the chest at any time in the past? (yes/no).” These questions were based on the validated International Study of Asthma and Allergies in Childhood (ISAAC) [18].

### Other variables

Covariates which may confound the relationship between timing of introduction to infant cereal and the outcomes were identified a priori based on a review of the literature. These included: breastfeeding duration, family history of asthma, child age, child sex, child’s season of birth, birth weight, gestational age, number of siblings, household smoking, parental ethnicity and reported family income.

Breastfeeding duration and family history of asthma were hypothesized as potential effect modifiers. Breastfeeding duration was dichotomized as < 4 mo or ≥ 4 mo to identify whether the child was breastfeeding when infant cereal was introduced. For children breastfed ≥ 4 mo and introduced to infant cereal at 6 mo, it is possible that they were not breastfeeding when infant cereal was introduced. Family history of asthma was defined as one or more biological parents or siblings with asthma. All exposure, outcome and other variables were collected at each TARGet Kids! visit.

### Statistical analysis

Descriptive analyses were performed to describe the primary exposure, outcomes and covariates from each child’s first visit. Categorical variables were expressed as counts and percentages, and continuous variables as mean and standard deviation (SD).

For the primary and secondary analyses, multinomial generalized estimating equation (GEE) models were used with a time-exchangeable correlation structure and robust variance measures [19]. All models included the exposure and covariates specified above. Additionally, all models accounted for possible non-linear effects of age. Outcome variables were treated as time-varying because eczema and wheezing may present at different times during childhood.

Time introduced to infant cereal was treated non-linearly in all analyses because existing literature suggests a non-linear relationship may exist [10–13]. Non-linearity was accounted for using restricted cubic splines (RCS) with 5 knots placed at pre-specified quantiles [20]. Pairwise comparisons using estimated marginal means were used to compare differences in estimated mean outcome values for introduction to infant cereal at 4 months vs. 6 months (the reference), as these time points for introduction of solid foods have been recommended by the AAP and CPS [7, 8, 21]. Pairwise comparisons using estimated marginal means creates comparisons from the fitted GEE models. A reference grid was created which includes the information needed to calculate the estimated marginal means, confidence intervals and make inferences on them. This is based on the variance–covariance matrix of the regression coefficient estimates [21]. Likelihood ratio tests (LRT) were used to determine if family history of asthma and breastfeeding duration were effect modifiers within each model. A single global test was used for effect modification and modifiers remained in the models if *P* < 0.30 [20]. For the primary and secondary analyses, model results were reported as odds ratios (OR) and 95% confidence intervals (CI).

All exposures, outcomes and covariates had ≤ 13% missing data. Multiple imputation was conducted using Multivariate Imputations by Chained Equations (MICE) with 20 imputed data sets to account for missing data with all covariates, outcomes and exposure in the models [22]. Auxiliary variables duration of formula feeding and volume of cow’s milk consumed were included to improve the multiple imputation model. Numeric data was imputed using predictive mean matching. Factor data with 2 levels was imputed with logistic regression imputation. Factor data with > 2 levels was imputed with polytomous regression imputation. Missing data were believed to be missing at random conditional on the variables in the imputation models. The variance inflation factor was < 2 for all covariates in each model. Results were considered statistically significant with *P* < 0.05. All statistical analyses were conducted using R version 4.0.2 [23].

## Results

**Fig. 1:**
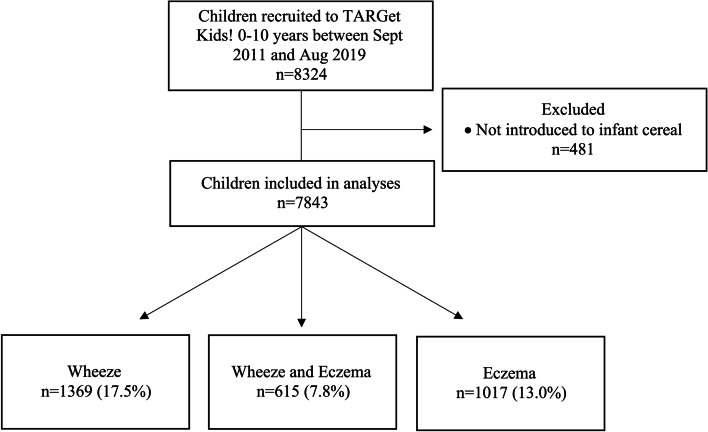
Participant flow chart. Legend: Bottom row shows number of children with eczema and/or wheeze. Child considered to be diagnosed with eczema or have wheezing if an affirmative response on the International Study of Asthma and Allergies in Childhood (ISAAC) questionnaire was provided at any visit. *n* = 4829 (61.6%) without wheeze or eczema

A total of 8324 children participated in TARGet Kids! between September 2011 and August 2019. After removing children who were not introduced to infant cereal (*n* = 481), 7843 children were included in the analyses (Fig. 1). The reasons for not introducing infant cereal were being too young to be introduced to solid foods (i.e. child’s only visit occurred prior to the introduction of solid foods) (*n* = 469) and introducing solid foods other than infant cereal (*n* = 12). Children were followed for 4.5 years on average, and 69% of children had at least 2 or more TARGet Kids! visits.

**Fig. 2:**
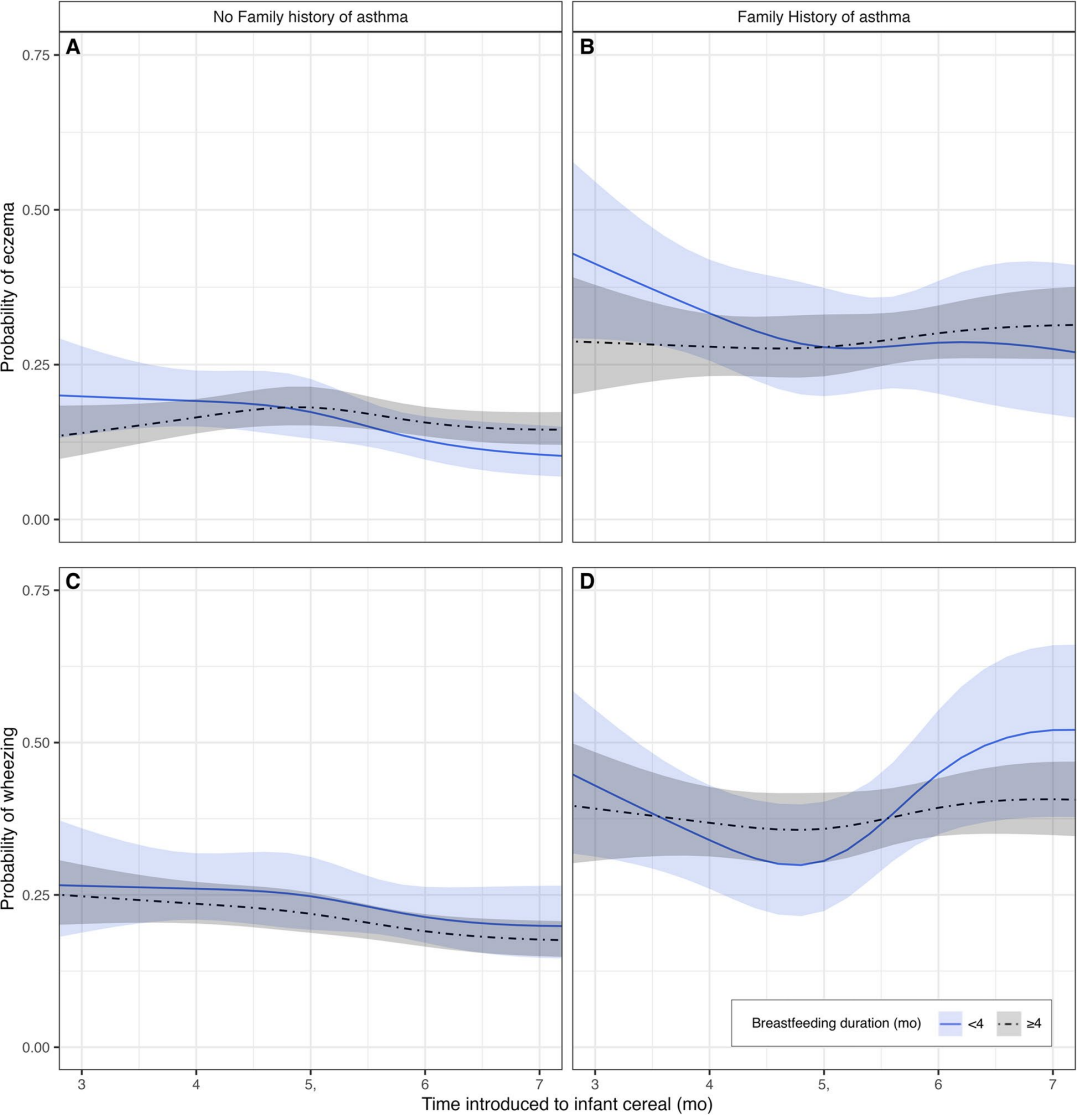
Timing of introduction to infant cereal and probability of eczema and wheezing by family history of asthma and breastfeeding duration. Legend: **A-D** Non-linear models adjusted for variables described in text. Shaded area shows 95% CI. **A** Probability of eczema for children without family history of asthma by breastfeeding duration. **B** Probability of eczema for children with family history of asthma by breastfeeding duration. **C** Probability of wheeze for children without family history of asthma by breastfeeding duration. **D** Probability of wheeze for children with family history of asthma by breastfeeding duration

The median age at the initial TARGet Kids! visit was 2 years and 52% of the children were male (Table 1). The mean (SD) age of introduction to infant cereal was 5.7 (1.9) months. 1386 (17.7%) children were introduced to infant cereal at 4 months and 3268 (41.7%) at 6 months of age. Fewer children who were introduced to infant cereal at 4 months were breastfed ≥ 4 months (76.2%) compared to those who were introduced to infant cereal at 6 months (85.6%). Otherwise, baseline characteristics appear to be similar for those introduced to infant cereal at 4 and 6 months of age. During the follow-up period, parent responses to the ISAAC questionnaire indicated that 13.0% of children were diagnosed with eczema, 17.5% had wheezing, and 7.8% had both eczema and wheezing. A child was considered to have eczema, wheeze, or both if an affirmative response was provided at any visit, as they may present at different times during childhood (Fig. 1). Figure 2 shows the association between the timing of introduction to infant cereal throughout the 4 to 6 month range and beyond and the probability of eczema and wheezing by family history of asthma and breastfeeding duration.

**Table 1:** Participant initial visit characteristics

**Child Characteristics**^**a**^	Time introduced to infant cereal (mo)		
	4 *n* = 1386	6 *n* = 3268	Total cohort *n* = 7843
Timing of introduction to infant cereal (mo)	4.1 (0.2)	6.0 (0.1)	5.7 (1.9)
Age at first visit (yr)	2.4 (2.4)	3.1 (2.4)	2.7 (2.4)
Child diagnosed with eczema or atopic dermatitis			
● Yes	185 (13.3)	435 (13.3)	1043 (13.3)
● No	1108 (79.9)	2608 (79.8)	6247 (79.7)
Child had wheezing			
● Yes	207 (14.9)	517 (15.8)	1188 (15.1)
● No	1159 (83.6)	2698 (82.6)	6517 (83.1)
Sex			
● Male	743 (53.6)	1706 (52.2)	4100 (52.3)
● Female	641 (46.2)	1557 (47.6)	3727 (47.5)
Birth weight (kg)	3.3 (0.6)	3.3 (0.6)	3.3 (0.6)
Gestational Age			
● 32–36 weeks	115 (8.3)	337 (10.3)	746 (9.5)
● 37 weeks	1125 (81.2)	2492 (76.3)	6091 (77.7)
Breastfeeding duration			
● < 4mo	292 (21.1)	363 (11.1)	1191 (15.2)
● ≥ 4mo	1056 (76.2)	2798 (85.6)	6397 (81.6)
Season of birth			
● Fall	372 (26.8)	792 (24.2)	1999 (25.5)
● Spring	349 (25.2)	839 (25.7)	1963 (25.0)
● Summer	350 (25.3)	864 (26.4)	2048 (26.1)
● Winter	315 (22.7)	773 (23.7)	1826 (23.3)
**Familial Characteristics**			
Number of siblings	0.8 (0.9)	0.9 (0.8)	0.9 (0.8)
Median	1.0	1.0	1.0
Household Cigarette Smoker			
● Yes	182 (13.1)	302 (9.2)	883 (11.3)
● No	1191 (85.9)	2926 (89.5)	6857 (87.4)
Family History of Asthma (Mother, Father, or Sibling)			
● Yes	233 (16.8)	542 (16.6)	1286 (16.4)
● No	963 (69.5)	2318 (70.9)	5506 (70.2)
Maternal Ethnicity			
● European	825 (59.5)	1900 (58.1)	4430 (56.5)
● Asian^b^	220 (15.9)	597 (18.3)	1418 (18.1)
● African	80 (5.8)	146 (4.5)	413 (5.3)
● Latin American	35 (2.5)	99 (3.0)	218 (2.8)
● Other (mixed, Arab)	109 (7.9)	241 (7.4)	633 (8.1)
Median after tax household income (CAD $)			
● 29,999	92 (6.6)	203 (6.2)	545 (6.9)
● 30,000–79,999	256 (18.5)	519 (15.9)	1365 (17.4)
● 80,000–149,999	442 (31.9)	1004 (30.7)	2396 (30.5)
● 150,000	547 (39.5)	1421 (43.5)	3222 (41.1)

^a^ Values reflect participant’s initial visit and are expressed as mean (SD) or n (%). Numbers may not add up to total due to missing values

^b^ East, South, and Southeast Asian

In the primary analysis, there was not strong evidence for an association between introduction to infant cereal at 4 vs. 6 months of age and higher odds of eczema in later childhood (OR 1.10; 95% CI: 0.97, 1.25; *P* = 0.14). However, at least one of family history of asthma or breastfeeding duration were effect modifiers (likelihood ratio test; *P* = 0.04). Introduction to infant cereal at 4 vs. 6 months of age was associated with higher odds of eczema in later childhood (OR 1.62; 95% CI: 1.12, 2.35; *P* = 0.01) among children without a family history of asthma who were not breastfeeding when solid foods were introduced. There was little evidence of an association between introduction to infant cereal at 4 vs. 6 months of age and higher odds of eczema in later childhood (OR 1.06; 95% CI: 0.90, 1.25; *P* = 0.47) among children without a family history of asthma who were breastfeeding when solid foods were introduced. Among those with a family history of asthma, there was not strong evidence for an association between introduction to infant cereal at 4 vs. 6 months of age and eczema whether the child was breastfed < 4mo (OR 1.25; 95% CI: 0.72, 2.18; *P* = 0.43) or ≥ 4mo (OR 0.91; 95% CI: 0.71 1.14; *P* = 0.39) (Table 2 and Fig. 2).

**Table 2:** Time introduced to infant cereal pairwise comparisons at 4 vs. 6 months and eczema and wheezing risk by family history of asthma and breastfeeding duration

Atopic Outcome	Family History of Asthma		No Family History of Asthma	
	Breast fed < 4mo **(OR (95% CI;* P*))** *n* = 287^**a**^	Breastfed ≥ 4mo **(OR (95% CI;* P*))** *n* = 1466^**a**^	Breast fed < 4mo **(OR (95% CI;* P*))** *n* = 834^**a**^	Breastfed ≥ 4mo **(OR (95% CI;* P*))** *n* = 4630^**a**^
Eczema^b^	1.25(0.72,2.18; 0.43)	0.91(0.71,1.14; 0.39)	1.62(1.12,2.35; 0.01)	1.06(0.90,1.25; 0.47)
Wheeze^b^	0.63(0.37,1.08; 0.10)	0.90(0.72, 1.13; 0.37)	1.29(0.93,1.81; 0.13)	1.31(1.13,1.52; < .001)

^a^ Numbers may not add up to total due to missing values

^b^ Models adjusted for birth weight, child age (RCS, 5 knots), child sex, child season of birth, gestational age, parental ethnicity, family income, household smoking, number of siblings and non-linear time introduced to infant cereal (RCS, 5 knots). Family history of asthma and breastfeeding duration included as effect modifiers. Introduction to infant cereal at 6 months is the reference group

In the secondary analysis, introduction to infant cereal at 4 vs. 6 months of age was associated with higher odds of wheezing in later childhood (OR 1.16; 95% CI: 1.03, 1.30; *P* = 0.01). At least one of family history of asthma or breastfeeding duration were effect modifiers (likelihood ratio test; *P* = 0.05). Introduction to infant cereal at 4 vs. 6 months of age was associated with higher odds of wheezing in later childhood (OR 1.31; 95% CI: 1.13, 1.52; *P* < 0.001) among children without a family history of asthma who were breastfeeding when solid foods were introduced. There was little evidence of an association between introduction to infant cereal at 4 vs. 6 months of age and higher odds of wheezing in later childhood (OR 1.29; 95% CI: 0.93, 1.81; *P* = 0.13) among children without a family history of asthma who were not breastfeeding when solid foods were introduced. Among those with a family history of asthma, there was not strong evidence for an association between introduction to infant cereal at 4 vs. 6 months of age and wheezing whether the child was breastfed < 4mo (OR 0.63; 95% CI: 0.37, 1.08; *P* = 0.10) or ≥ 4mo (OR 0.90; 95% CI: 0.72, 1.13; *P* = 0.37) (Table 2 and Fig. 2).

## Discussion

In this longitudinal cohort study, we evaluated the relationship between the timing of introduction to infant cereal at 4 months compared to 6 months of age and parent reported eczema or wheeze, and whether these relationship were modified by family history of asthma and breastfeeding duration. We found evidence for family history of asthma and breastfeeding duration to be effect modifiers in each of the models. In particular, among children without a family history of asthma, introduction to infant cereal at 4 months compared to 6 months of age was associated with higher odds of eczema for children who were not breastfeeding when solid foods were introduced and higher odds of wheezing for children who were breastfeeding when solid foods were introduced. There was little evidence of associations among the remaining strata. Taken together, these findings support the current AAP and CPS recommendations for introducing solid foods around 6 months of age among infants who are at low risk for allergy [7, 8, 24, 25].

Our analysis focused on comparing the introduction of solid food at 4 months to 6 months, as these are the times currently recommended by professional organizations for optimal health. We used the validated ISAAC questionnaire to measure parent reported wheezing, as asthma diagnosis in young children is challenging, and wheezing is an important outcome. Eczema diagnosis, on the other hand, is straightforward so parent reported diagnosis of eczema was chosen. Previous studies have shown that the introduction to solid food < 4 [10, 13] months or > 6 [11, 12] months was associated with higher risk of eczema or wheeze. Taylor-Robinson et al. found that children participating in the United Kingdom Millennium Cohort Study who were introduced to solid food < 4 months had higher odds of eczema by 5 years of age compared to those introduced ≥ 4 months (*n* = 14,499) [10]. Conversely, Snijders et al. found that children participating in the Netherlands KOALA Birth Cohort Study who were introduced to solid food > 7 months had higher odds of AD, eczema and wheeze by 2 years of age compared to those introduced at 4 to 6 months (*n* = 2,558) [11]. A recent systematic review conducted by Obbagy et al. as part of the Pregnancy and Birth to 24 Months Project found moderate evidence of no relationship between the timing of introduction to solid food and AD or asthma [26]. However, the authors noted a number of limitations of the existing literature including lack of adjustment for important confounding variables (e.g. breastfeeding status, parental smoking), non-representative populations (which mainly included subjects at higher risk for atopic disease) and short duration of follow-up [26]. To our knowledge no previous study has examined the relationship between solid food introduction within the 4 to 6 month range and eczema and wheezing.

In the present study, we found that earlier introduction to solid food (i.e. 4 months compared to 6 months) was less favourable for the presence of eczema or wheezing among those without a family history of asthma. We speculate that introducing solid foods earlier, when the microbiota and immune system are less mature, may affect the risk of later atopic disease [27]. The microbiota rapidly develops during infancy and resembles the adult microbiota as early as 1 year of age [28]. The colonization of the microbiota plays an important role in immunity. For instance, microbes contribute to the intestinal barrier function and the microbiota can induce regulatory T-cell production which help to supress an immune response (i.e. achieve oral tolerance) [27, 28].

We hypothesized that the relationship between the timing of introduction to infant cereal and eczema and wheeze might be modified by family history of asthma and breastfeeding duration. Later introduction of solid foods can extend the duration of exclusive breastfeeding. Breastfeeding has been hypothesized to be protective of eczema or wheezing by modulating the microbiota and the immune system [15, 27]. Breastmilk offers a number of anti-inflammatory compounds and supports host defence mechanisms within the gastrointestinal tract where new antigens are encountered [29]. Food antigens are also transferred through breastmilk from the mothers diet. This may be beneficial in inducing tolerance, but potentially consequential because of the immature intestinal barrier [29]. In the present study, breastfeeding duration modified the association between the timing of introduction to infant cereal in opposite directions for eczema and wheezing. Prior research has found inconsistent relationships between breastfeeding and eczema and wheezing [14, 15]. For instance, Kull et al. found that children in a prospective birth cohort study (BAMSE) in Sweden who were exclusively breastfed ≥ 4 months, compared to < 4 months, had lower risk for eczema at 4 years of age (*n* = 4089) [30]. On the other hand, Taylor-Robinson et al. found that among children in the United Kingdom Millennium Cohort Study, longer exclusive breastfeeding duration was associated with higher risk of eczema [10]. In the present study the odds for wheezing was similar among those breastfed < 4mo vs. ≥ 4mo.

Among children with a family history of asthma, we did not find strong evidence for an association between the timing of introduction to solid foods and eczema or wheezing regardless of breastfeeding duration. Family history of allergy may be such a potent risk factor for the development of atopic disease that it may not be sensitive to differences in the timing of introduction to solid food between 4 and 6 months. Alternatively, the timing of introduction to allergenic solid foods may be of greater impact, particularly for food allergy prevention [31]. For children at high risk for allergy (e.g. have a family history of allergy), the AAP and CPS recommend introducing allergenic solid foods between 4 and 6 months of age [24, 25]. This recommendation is based upon several randomized trials which have included children at high risk for allergy and found a reduction in food allergy when allergenic solid foods (particularly peanut and egg) are introduced as early as 4 months [24, 25]. In Canada, the CPS has only recommended introducing allergenic solid foods among high risk infants as early as 4 months since 2019, prior to data collected in this study and thus, we did not explore calendar date within our analyses. Children without a family history of asthma may be more sensitive to environmental changes such as the timing of introduction to solid food.

Strengths of this study include the longitudinal design with repeated measures among a large, ethnically diverse cohort of healthy urban children. Validated questionnaire data permitted evaluation of multiple clinically relevant outcomes with adjustment for important potentially confounding variables. Detailed clinically relevant data helped us to understand the complexity of solid food introduction by exploring how family history of asthma and breastfeeding duration modified the relationship between the timing of introduction to infant cereal within the 4 to 6 month age range and eczema and wheezing.

There are several limitations to this study. Due to the observational nature of this study, there is the potential for unmeasured confounding, so causality cannot be established. For example, due to data limitations, we were unable to account for family history of some atopic diseases such as eczema. Timing of introduction to infant cereal was used as a proxy for solid food introduction and was measured retrospectively by parent report which may be subject to recall bias. Reverse causality is also a possibility. For example, parents of children who developed early atopic disease symptoms may delay introducing solid foods and breastfeed longer. However, all models included adjustment for baseline differences in eczema and wheezing prior to the introduction to solid food, when available. Lastly, while TARGet Kids! includes an ethnically diverse sample of urban children, it may not be representative of children in other settings, generalizable to children with chronic health conditions or applicable to infants who were not introduced to infant cereal as a first solid food.

## Conclusion

In this longitudinal cohort study of healthy children followed through 10 years of age, introduction to solid food at 4 months compared to 6 months of age was associated with a higher risk of eczema or wheezing in later childhood among children without a family history of asthma. These findings align with current AAP and CPS recommendations for introducing solid foods around 6 months of age for children at low risk of allergy.

## Data Availability

The datasets used and/or analysed during the current study are not publicly available to maintain participant confidentiality but are available from the corresponding author on reasonable request.

## Abbreviations

AAP: American Academy of Pediatrics
AD: Atopic Dermatitis
CPS: Canadian Paediatric Society
ESPGHAN: European Society for Paediatric Gastroenterology, Hepatology, and Nutrition
GEE: Generalized Estimating Equation
ISAAC: International Study of Asthma and Allergies in Childhood
LRT: Likelihood Ratio Test
RCS: Restricted Cubic Splines
